# Serum Activities of Ferritin Among Controlled and Uncontrolled Type 2 Diabetes Mellitus Patients

**DOI:** 10.7759/cureus.25155

**Published:** 2022-05-20

**Authors:** Sarat Chandan Tummalacharla, Pratyusha Pavuluri, Shravya Reddy Maram, Sabitha Vadakedath, Deepthi Kondu, Soujanya Karpay, Venkataramana Kandi

**Affiliations:** 1 Biochemistry, RVM Institute of Medical Sciences and Research Centre, Siddipet, IND; 2 Medicine, RVM Institute of Medical Sciences and Research Centre, Siddipet, IND; 3 Biochemistry, Prathima Institute of Medical Sciences, Karimnagar, IND; 4 Clinical Microbiology, Prathima Institute of Medical Sciences, Karimnagar, IND

**Keywords:** type 2 diabetes mellitus, hyperferritinemia, ferritin, uncontrolled diabetes, controlled diabetes

## Abstract

Background

Diabetes mellitus (DM) is a metabolic disorder characterized by the cells' inefficient utilization of blood glucose. DM occurs in two types: type 1 DM (T1DM) and type 2 DM (T2DM). DM results in increased blood sugar levels attributed to the non-functioning of the insulin-producing islet cells of the pancreas (type 1 DM) and insulin resistance, among other causes. Despite the initiation of treatment, in some people, diabetes remains uncontrolled and, over some time, could cause damage to other organs of the body, including the eyes, heart, and kidneys, among others. Recently, it was observed that iron metabolism and increased activity of serum ferritin (hyperferritinemia) could influence the development of T2DM. This study aims to assess the activities of ferritin among controlled and uncontrolled T2DM patients and compare them with the control group who were non-diabetic.

Methods

The study included 30 controlled and uncontrolled T2DM patients and an equal number of controls. The study was conducted between September and October 2021, and all patients included were those attending the General Medicine outpatient department attached to the RVM Institute of Medical Sciences and Research Centre, Siddipet, Telangana, South India. Blood glucose activities were estimated by the glucose oxidase-peroxidase (GOD-POD) method using the Randox Daytona plus analyzer, and serum ferritin was measured by the chemiluminescence method using the Beckmann Coulter Access 2 instrument.

Results

The mean age of the cases and the controls was 56.5 years and 46.7 years, respectively. Serum ferritin activities among people with controlled diabetes (73.3±56.6 ng/ml) (p=0.0003) and uncontrolled diabetes (269.8±347.1 ng/ml) (p=0.0006) varied significantly as compared to the controls (40.853±15.55). Glucose activities among controls (82.9±7.4 mg/dl), controlled T2DM patients (120.9±28.6 mg/dl), and uncontrolled T2DM patients (316.06±145.41 mg/dl) also showed significant differences.

Conclusion

Hyperferritinemia is evident among uncontrolled T2DM patients. However, increased serum ferritin activities were also noted among controlled T2DM patients as compared to normal activities observed in the non-diabetic control group.

## Introduction

Diabetes is an endocrine disorder related to the abnormal activity of insulin secreted by the islets of the pancreas. Insulin is essential for the absorption of glucose into the cells, and when its activities are compromised for various reasons, people suffer from increased levels of glucose in the blood, which is called hyperglycemia. Therefore, diabetes is generally diagnosed by detecting hyperglycemia in the patient's blood. Diabetes occurs in two types: type 1 diabetes results from the inability of the islets to secrete insulin, and in most instances, it is attributed to genetic causes and is hereditary. Type 2 diabetes mellitus (T2DM) is a condition wherein insulin secretion becomes inadequate and is generally a disease of aging. However, T2DM can occur at a much earlier age and is multifactorial. T2DM is characterized by insulin resistance, decreased glucose utilization, increased glucose accumulation, and impaired insulin secretion. The predisposing factors for T2DM include dormant lifestyles, excessive sugar consumption, obesity, and genetic predisposition, among others. The management of DM involves intravenous insulin injections and other medications that can be taken orally. Improperly managed DM can result in complications in other organs of the body, including the eyes, kidneys, and heart. Despite the availability of effective therapeutic interventions to control DM, in some people, the blood glucose levels do not completely return to the normal range, and this condition may be labeled as uncontrolled diabetes.

Evidence has been emerging about the relationship between iron metabolism and T2DM. It was presumed that there could be a bi-directional relationship wherein iron metabolism impacts glucose metabolism and vice versa. It was also suggested that diminished activities of iron can affect insulin secretion and thereby help control T2DM [1]. Other factors that could be responsible for the dysregulation of iron and glucose metabolism include diet, imbalances in the synthesis of heme and absorption of iron, and liver function, among others [2]. A recent meta-analysis has also confirmed the potential role of serum ferritin along with hepcidin in the development of T2DM [3]. The excessive dietary intake of heme iron was also noted to be responsible for the development of T2DM [4]. Moreover, it was observed that adherence to a particular diet that may potentially restrict high iron intake can lower the risk of T2DM [5].

Given the pieces of evidence available in the literature that indicated the association of iron metabolism with T2DM, we in this study have attempted to assess the activities of serum ferritin among the controlled and uncontrolled T2DM patients and compared them with the control group who did not have T2DM.

## Materials and methods

This is an analytical cross-sectional study and included 30 controlled T2DM patients, 30 uncontrolled T2DM patients, and 30 healthy and non-diabetic controls. It was carried out between September and October 2021. All the patients included in the study were attending the General Medicine outpatient department attached to the RVM Institute of Medical Sciences and Research Centre, Telangana, South India. The study was approved by the institutional ethics committee, and all the study participants have voluntarily given their consent to participate in the study.

Inclusion and exclusion criteria

All the subjects in the age group of 45-60 years, including both males and females, who were diagnosed with T2DM and T2DM patients without any prior complications, were recruited as the study group. All patients who were tested and found to be non-diabetic and were asymptomatic and healthy were recruited into the control group. Patients under 45 years old or over 60 years old who were not willing to give their consent to participate, patients with a high C-reactive protein (CRP), patients who had blood transfusions or iron supplements, and patients with complications from diabetes like nephropathy, retinopathy, and neuropathy were excluded from the study.

Five milliliters of venous blood were collected from each participant. Fasting blood glucose, hemoglobin, serum ferritin, and glycated hemoglobin (HbA1c) were estimated by using standard protocols. Also, 1 ml of venous blood was collected from the cases after two hours of an oral glucose tolerance test (OGTT) and/or postprandial for the estimation of blood glucose. Blood glucose was estimated by the glucose oxidase-peroxidase (GOD-POD) method on a Randox Daytona plus analyzer (Randox Laboratories Ltd., Crumlin, UK). Serum ferritin was measured by the chemiluminescence method in a Beckmann Coulter Access 2 instrument (Beckman Coulter, Inc., Brea, CA, USA). HbA1c and blood hemoglobin were estimated by using high-performance liquid chromatography (HPLC) and an automated analyzer, respectively.

Criteria used for the diagnosis of T2DM

The criteria used for the diagnosis of T2DM include an HbA1c of >6.5%, a fasting blood glucose of >126 mg/dl, two-hour blood glucose of >200 mg/dl after an OGTT, and/or post-prandial.

Criteria used to identify uncontrolled T2DM patients

All T2DM patients who have been taking diabetes medication and present with abnormalities of HbA1c, fasting blood glucose, and post-prandial are identified as uncontrolled T2DM patients.

Statistical analysis

The collected data were entered into Microsoft Excel 2019 sheet (Microsoft® Corp., Redmond, WA), and SPSS software version 21 (IBM Corp., Armonk, NY) was used for the preparation of tables and to perform statistical analysis. Descriptive statistics of the three groups of patients were demonstrated as mean and standard deviation. A p-value of <0.05 was taken as significant.

## Results

Serum ferritin activities among controlled, uncontrolled T2DM patients, and the control group were 73.3±56.6 ng/ml, 269.8±347.1 ng/ml, and 40.853±15.55 ng/ml, respectively. In comparison, serum ferritin activity in the control group varied significantly (p=0.0037) from the controlled T2DM patients. A significant (p=0.0006) variation was also observed with the activities of serum ferritin in the control group and uncontrolled T2DM patients. Also, the serum ferritin activity varied significantly (p=0.0033) among the controlled T2DM patients and uncontrolled T2DM patients.

The blood glucose levels among the control group (82.9±7.4mg/dl), controlled T2DM patients (120.9±28.6 mg/dl), and uncontrolled T2DM patients (316.06±145.41 mg/dl) revealed considerable variations. The blood hemoglobin levels among the control group, controlled T2DM, and uncontrolled T2DM patients were 14.58±1.48 g/dl, 13.80±1.81 g/dl, and 14.05±1.80 g/dl, respectively. The activities of HbA1c among the control group, controlled and uncontrolled T2DM patients were 4.28%, 6.12%, and 10.4%, respectively. The activities of serum ferritin, blood glucose, HbA1C, and hemoglobin among various study groups are shown in Table 1.

**Table 1:** The activities of ferritin, blood glucose, HbA1C, and hemoglobin among various study groups T2DM: Type 2 diabetes mellitus; HbA1C: glycated hemoglobin; *statistically significant

Subjects/parameter	Ferritin (mean±SD) (ng/ml)	Blood glucose (mean±SD) (mg/dl)	HbA1C (mean±SD) (%)	Hemoglobin (mean±SD) (g/dl)
Controls	40.83±15.55	82.9±7.4	4.28±0.50	14.58±1.48
Controlled T2DM	73.30±56.6	120.9±28.6	6.12±0.46	13.80±1.81
Uncontrolled T2DM	269.8±347.1	316.06±145.41	10.4±0.80	14.05±1.80
p-value	= 0033*	<0.0001*	<0.0001*	= 0.5937

## Discussion

DM is the most prevalent non-communicable disease responsible for morbidity and mortality throughout the world. India stands second only to China in terms of the global burden of diabetes, with more than 77 million people affected by DM. Reports have also suggested that more than 50% of DM cases remain undiagnosed both in India and across the world [6]. Despite the availability of effective therapeutic interventions to treat and manage DM, several people suffer from uncontrolled blood glucose levels [7]. The cause of uncontrolled DM is multifactorial and includes dietary control, physical activity, and insufficient self-care behavior among people with DM, among several others [8]. The roles of vitamins (water-soluble and fat-soluble), minerals (chromium, zinc, selenium, potassium), and amino acids (leucine, taurine, beta-alanine) have recently been proposed, along with other dietary macronutrients and fiber that may potentially cause insulin resistance and impaired glucose metabolism and contribute to the development of uncontrolled DM [9,10].

Ferritin is a protein that facilitates the storage and sequestration of iron and protects cells from its toxicity. Ferritin is present in almost all human cells, and it acts as an iron reserve that is readily available for the formation of hemoglobin and other heme proteins. The expression of ferritin is tightly regulated at the transcriptional and posttranscriptional levels by a variety of factors, including iron, hormones, cytokines, and oxidative stress, among others. The serum ferritin concentration roughly echoes the body's iron content, and it also acts as a positive acute-phase reactant in various inflammatory conditions. However, hyperferritinemia is not synonymous with iron overload, and many disorders result in increased serum ferritin concentration that does not always correlate with an elevation in the body's iron content. Elevated ferritin was previously described in infections, hyperthyroidism, liver failure, renal failure, chronic alcohol consumption, rheumatic disease, inflammatory disease, malignancy, and metabolic syndrome, among others [11-13].

The role of iron metabolism and its relationship with metabolic syndrome and T2DM has recently gained increased attention. Iron overload was found to be particularly associated with the development of metabolic syndrome and related disorders [14]. Iron plays an important role in the functioning of human cells and any disturbances in its homeostasis will result in iron overload and deficiencies [15]. The serum concentrations of ferritin were significantly higher among T2DM patients as compared with the control group (227 (140-352) vs 203.5 (130.5-312) ng/mL, p < 0.05) in a Chinese study [16]. These results were in concordance with the results of the present study. This study supports the understanding that T2DM facilitates a proinflammatory environment that stimulates hepcidin. Hepcidin in turn activates transferrin, resulting in iron overload, causing insulin resistance and impaired glucose tolerance.

An increase in the activity of serum ferritin among nondiabetic people was found to correlate with impaired glucose tolerance. Therefore, it was suggested that hyperferritinemia could cause insulin resistance and hyperferritinemia individuals are predisposed to developing T2DM [17].

Studies have demonstrated the role of blood donation and iron restriction in improving insulin secretion and glucose metabolism [18,19]. It was also noted that treatment with dapagliflozin, an SGLT2 inhibitor, contributed to reduced iron overload, increased hemoglobin, and reduced HbA1C activities [20].

## Conclusions

The results of the present study suggest that iron homeostasis is critical both in the control and development of T2DM. Moreover, a few studies in the past have also indicated the role of hyperferritinemia as a potential cause of metabolic disorders, including T2DM. Therefore, regular monitoring of serum ferritin activity among people who could be predisposed to T2DM assumes increased significance. It is also essential to monitor the serum ferritin and blood glucose levels among T2DM patients who are currently undergoing therapy to prevent the development of T2DM-related complications. Restricting the nutritional intake of iron and prescribing therapeutic drugs to minimize the activity of iron may prove beneficial in the control and management of T2DM. Furthermore, clinical trial studies are necessary to evaluate the utility of iron-reducing therapeutics in the prevention, control, and management of T2DM.
